# Optimizing antifungal dosing for invasive *Cryptococcus* infections: minimum inhibitory concentration distributions and pharmacokinetic/pharmacodynamic insights from 2010–2023 Antimicrobial Testing Leadership and Surveillance data

**DOI:** 10.3389/fphar.2025.1665253

**Published:** 2025-10-10

**Authors:** Chia-Ying Liu, Chih-Cheng Lai, Chun-Chung Hsueh, Chih-Jen Weng, Wei-Lun Chang, Po-Ren Hsueh, Shio-Shin Jean

**Affiliations:** ^1^ Department of Infectious Diseases and Department of Hospitalist, Far Eastern Memorial Hospital, New Taipei City, Taiwan; ^2^ General Education Center, Lunghwa University of Science and Technology, New Taipei City, Taiwan; ^3^ Department of Intensive Care Medicine, Chi Mei Medical Center, Tainan, Taiwan; ^4^ School of Medicine, College of Medicine, National Sun Yat-sen University, Kaohsiung, Taiwan; ^5^ Department of Internal Medicine, Taipei Veterans General Hospital, Taipei, Taiwan; ^6^ Division of Nephrology, Department of Internal Medicine, Min-Sheng General Hospital, Taoyuan, Taiwan; ^7^ Department of Pharmacy, Far Eastern Memorial Hospital, New Taipei City, Taiwan; ^8^ Departments of Laboratory Medicine and Internal Medicine, China Medical University Hospital, China Medical University, Taichung, Taiwan; ^9^ Departments of Laboratory Medicine and Internal Medicine, National Taiwan University Hospital, National Taiwan University College of Medicine, Taipei, Taiwan; ^10^ Department of Pharmacy, College of Pharmacy and Health Care, Tajen University, Pingtung, Taiwan; ^11^ Departments of Internal Medicine and Critical Care Medicine, Min-Sheng General Hospital, Taoyuan, Taiwan

**Keywords:** *Cryptococcus* species, fluconazole, voriconazole, posaconazole, isavuconazole, liposomal amphotericin B

## Abstract

**Objective:**

The 2024 global cryptococcosis treatment guidelines suggests that fluconazole (FLC) combined with liposomal amphotericin B (AMB) and 5-flucytosine (5-FC) as the mainstay of treatment for systemic cryptococcosis. Although this 2024 guidelines also list recommend voriconazole (VRC), posaconazole (POS), and isavuconazole (ISA) as alternatives to FLC during the consolidation and maintenance phases, current data on distributions of minimum inhibitory concentrations (MICs) of global *Cryptococcus* isolates for antifungals—and studies evaluating the application of their pharmacokinetic (PK) profiles and pharmacodynamic (PD) indices in the treatment of systemic cryptococcosis—remain limited. To optimize antifungal dosing, integration of global MIC distributions for *Cryptococcus* isolates with PK/PD parameters for key antifungal agents is needed.

**Methods:**

This study analyzed the MIC distributions from the 2010–2023 antifungal Antimicrobial Testing Leadership and Surveillance database, and determined epidemiological cutoff values for major *Cryptococcus* species.

**Results:**

The majority of invasive *Cryptococcus* isolates were classified as wild-type strains (>90%). We analyzed PK profiles (particularly central nervous system [CNS] penetration from the bloodstream), PD indices of antifungals (azoles and AMB) against yeasts. Based on 25 studies clearly describing PK–PD relationships, FLC and VRC were considered optimal choices because of superior CNS penetration. The optimal dose of FLC is 800–1,200 mg/day, whereas dosages of VRC and ISA do not require adjustment. Nevertheless, therapeutic drug monitoring for VRC is warranted during its prescription due to significant variability in plasma concentrations influenced by multiple factors. POS is not suitable for induction therapy in systemic cryptococcosis. Additionally, ISA is preferred over POS for consolidation therapy for *Cryptococcus* meningitis/meningoencephalitis (MME) based on differences in their PK profiles. Furthermore, a single 10 mg/kg dose of liposomal AMB—a cost-effective strategy—should be combined with 1,200 mg/day FLC and 5-FC, or alternatively VRC, as an effective induction-phase regimen for treating *Cryptococcus* MME.

**Conclusion:**

Diverging from the 2024 guidelines, this study provides novel insights into the treatment of *Cryptococcus* MME based on MIC distributions and PK-PD indices for antifungal agents.

## Introduction

Cryptococcal meningitis and meningoencephalitis (MME) are major causes of global infectious morbidity and mortality and can affect patients regardless of immune status (Iyer et al., 2021; Lee et al., 1996; Sudan et al., 2013; Su et al., 2024). Mortality rates due to cryptococcal MME reportedly range from 24% to 47% at 10 weeks (Jarvis et al., 2022; Molloy et al., 2018). In 2022, the World Health Organization listed *Cryptococcus neoformans* as a top fungal priority pathogen (Chang et al., 2024; World Health Organization, 2022).

Fluconazole (FLC) has been widely used for over 3 decades in the treatment of fungal infections. However, its increased global use has been associated with rising minimum inhibitory concentrations (MICs) against a variety of fungal pathogens (Bicanic et al., 2007; Pfaller et al., 2009; Sar et al., 2004). Due to FLC’s relatively narrow antifungal spectrum and the gradual increase in resistance, alternative antifungal agents with broader activity have been developed over the past 2 decades to target clinically important fungal species (Su et al., 2024).

Infections caused by *Cryptococcus* species in the central nervous system (CNS) are not confined to the cerebrospinal fluid (CSF). Lee et al. reported that these infections not only result in severe meningitis due to disruption of the blood-CSF barrier, but also involve the brain parenchyma, leading to severe encephalitis (Lee et al., 1996). This is particularly common in immunocompromised hosts such as patients with chronic human immunodeficiency virus (HIV) infection, Hodgkin’s disease, liver cirrhosis, or those undergoing solid organ transplantation (SOT), etc. (Chang et al., 2024). Therefore, when selecting antifungal agents for the treatment of CNS cryptococcosis, it is essential to consider their penetration into both the CSF and brain tissue. FLC and voriconazole (VRC) have demonstrated effective CNS penetration (see below), even in the absence of meningeal inflammation—unlike amphotericin B (AMB) and posaconazole (POS) (Felton et al., 2014; Nau et al., 2010).

The 2024 guidelines for cryptococcosis treatment recommend all newer triazoles as alternative agents (Chang et al., 2024). Although the guidelines mention alternative triazoles, robust evidence on MIC distributions and pharmacokinetic (PK)/pharmacodynamic (PD) analyses for *Cryptococcus* are scarce. Comodeling both PK and PD data—where available—provides valuable insights into the importance of tissue concentrations for antifungal agents (Felton et al., 2014). This knowledge gap, therefore, prompted us to analyze the variability among these key antifungal agents from multiple perspectives relevant to the effective treatment of cryptococcal infections worldwide.

The variability of MIC measurements of microorganisms—including *Cryptococcus* isolates and other fungi—against a given antifungal is well recognized. This variability is generally attributed to biological variation (i.e., inter-strain differences), intra-laboratory assay variability, and inter-laboratory methodological differences (Espinel-Ingroff et al., 2009; Espinel-Ingroff et al., 2012a; Mouton et al., 2018). Nevertheless, at any site of infection, MIC data for a specific antimicrobial against pathogens are crucial for understanding exposure-response relationships. Additionally, when the PK-PD parameters are applied, it is also important to consider that the hysteresis phenomenon, which refers to a delayed distribution of an antimicrobial to the site of action or the involvement of active metabolites. This may result in a lag or persistence in the antimicrobial effect (liposomal AMB [LAmB] (Bekersky et al., 2000) and caspofungin), even when the plasma drug concentration has declined (Felton et al., 2014; Hope, 2012).

The epidemiological cutoff values (ECVs) of antifungal agents against *Cryptococcus* species are not predictive of *in vivo* efficacy (Pharkjaksu et al., 2020), but they are used to distinguish wild-type (WT) from non-WT (i.e., resistant) isolates (CLSI, 2018). Due to the complexity of interpreting susceptibility data, we referred to the ECVs of antifungals against *Cryptococcus* species documented in the PubMed database (Jean et al., 2024), rather than relying on a single MIC value—such as MIC_90_ values of invasive *Cryptococcus* isolates for given antifungals—as a surrogate for further analysis.

Given the known PK variability of agents such as VRC, incorporating ECV-based strategies for dose optimization is methodologically sound—particularly in the absence of robust PD targets for *Cryptococcus* infections. In this context, PD targets established for *Candida* species (Li et al., 2010) are cautiously extrapolated to *Cryptococcus* isolates in this investigation. Furthermore, relatively few studies have estimated concentration-time profiles of antifungals in humans. Therefore, PK data for key antifungal agents—including data from laboratory animal models, where relevant and where corresponding human data are unavailable—were obtained from previously published sources (Jean et al., 2022). In this study, we applied the documented ranges of PK parameters, rather than relying solely on mean values, to evaluate the probability of PK/PD target attainment. The novelty of this study, which combines Antimicrobial Testing Leadership and Surveillance (ATLAS) MIC data with PK/PD analysis, lies in its indirect assessment of the appropriateness of various antifungal dosages for treating *Cryptococcus* infections, which often involve the CSF and cerebral tissue.

## Materials and methods

### Species, antifungal MICs, and MIC distributions of *Cryptococcus* isolates

Pfizer Pharmaceuticals (New York City, NY, USA) conducted the ATLAS project to investigate the MIC data of clinically significant microorganisms involved in global clinical infections to indicated antibiotics since 2006. To investigate the MIC distributions of important *Cryptococcus* species to the key antifungal agents, we extracted the antifungal 2010–2023 ATLAS database for analysis. The *Cryptococcus* isolates for which MIC data were determined for isavuconazole (ISA) were available since 2017.

The majority of *Cryptococcus* isolates were collected from blood or CSF samples. Additionally, the antifungal MIC profiles of *Cryptococcus* isolates collected from other clinical specimens—such as aspirated fluid from cysts or intra-articular space, wound pus, pleural or peritoneal fluid, biopsied tissue from the gastrointestinal tract, urinary tract, sinus, head, neck, or face—were also analyzed in patients with cryptococcosis worldwide. Antifungal MICs against the *Cryptococcus* isolates obtained directly from human brain tissue were not available in the ATLAS database. Isolates collected from other sources were excluded from further analysis.

Standard biochemical tests were used to accurately identify *Cryptococcus* species in each hospital participating in the ATLAS project. All isolates of the *C. gattii* species complex (hereafter referred to as *Cryptococcus gattii*) were further confirmed by examining melanin production on 3,4-dihydroxyphenylalanine media (Kwon-Chung et al., 1982). Additionally, DNA sequences from multiple genes, such as URA5, LAC1, CAP59, and CAP64, as well as the intergenic spacer and the internal transcribed spacer regions of the ribosomal DNA gene cluster, were used as supplementary methods to distinguish species within *C. neoformans* var. *neoformans* (hereafter referred to as *C. neoformans*), *C*. *neoformans* var. *grubii* (hereafter referred to as *Cryptococcus grubii*) and *C. gattii* (Chen et al., 2014).

Broth microdilution testing was used to determine the MIC levels of *Cryptococcus* isolates to the key antifungal agents (AMB, FLC, VRC, POS, and ISA). The tests were conducted according to the protocol M27-A4 of the Clinical and Laboratory Standards Institute. RPMI 1640 medium containing 0.2% glucose was used, and an inoculum ranging from 0.5 × 10^3^ to 2.5 × 10^3^ cells/mL was incubated in the air at 35 °C (CLSI, 2017). The concentration range tested in this study was 0.004–64 mg/L.

### Data synthesis

The MIC distributions of all studied *Cryptococcus* isolates—regardless of whether they were sourced from bloodstream infections (BSI) or CSF—as well as those of the three predominant *Cryptococcus* species for key antifungal agents under investigation, were clearly presented in the respective tables. Additionally, correlation analyses of MIC distributions among subsets of *Cryptococcus* isolates for different triazoles were presented in the corresponding figures.

### Statistical analyses

The MIC distributions of isolates of different *Cryptococcus* species for a specific antifungal agent were compared using the Mann-Whitney U test, where appropriate. Additionally, correlation analysis was performed to evaluate the relationships in MIC trends among invasive *Cryptococcus* isolates causing BSI and MME for the four azole agents, as appropriate. All statistical calculations were two-tailed, and a *P* value less than 0.05 was considered statistically significant. All statistical analyses were conducted using SPSS version 17.0 (IBM Corp., Armonk, NY, USA).

## Results

### MIC distributions and non-WT proportions of *Cryptococcus* isolates for different antifungals

Of all 488 *Cryptococcus* isolates under evaluation between 2010 and 2020, 395 (80.9%) were obtained from blood (*n* = 201) or CSF (*n* = 194), with the remaining 93 isolates (19.1%) originating from various other specimens.

The MIC distributions for *Cryptococcus* isolates, whether sourced from bloodstream or MME (as indicated by CSF cultures), exhibited a high degree of congruence across all tested antifungals (Table 1). Additionally, we used the Mann–Whitney U test to compare the MIC distributions of *Cryptococcus* isolates cultured from miscellaneous specimens with those causing BSI/MME against key antifungal agents (Table 2). This analysis revealed similar susceptibility patterns among *Cryptococcus* isolates from various infection sources.

**TABLE 1:** Distributions of minimum inhibitory concentrations (MICs) for 395 *Cryptococcus* isolates cultured from blood (*n* = 201) or cerebrospinal fluid (CSF; *n* = 194), tested against key antifungal agents. Isolates were collected from hospitalized patients with cryptococcosis worldwide, based on data from the antifungal Antimicrobial Testing Leadership and Surveillance between 2010 and 2020. Numerals in parentheses indicate the cumulative percentage of *Cryptococcus* isolates inhibited at each respective MIC value (mg/L) for a given antifungal agent.

MICs (mg/L) of tested BSI and CSF *Cryptococcus* isolates to different antifungals (no. of isolates)	No. (cumulative percentage) of isolates with indicated MIC values (mg/L)													MIC_50_	MIC_90_
	0.004	0.008	0.015	0.03	0.06	0.12	0.25	0.5	1	2	4	8	16		
AMB (395)						1 (0.3)	19 (5.1)	148 (42.5)	224 (99.2)	3 (100)				1	1
BSI (201)							10 (5.0)	77 (43.3)	114 (100)					1	1
CSF (194)						1 (0.5)	9 (5.2)	71 (41.8)	110 (98.5)	3 (100)				1	1
FLC (395)							2 (0.5)	11 (3.3)	55 (17.2)	143 (53.4)	147 (90.6)	34 (99.2)	3 (100)	2	4
BSI (201)							1 (0.5)	4 (2.5)	30 (17.4)	76 (55.2)	71 (90.5)	17 (99.0)	2 (100)	2	4
CSF (194)							1 (0.5)	7 (4.1)	25 (17.0)	67 (51.5)	76 (90.7)	17 (99.5)	1 (100)	2	4
VRC (395)		10 (2.5)	53 (15.9)	162 (57.0)	138 (91.9)	25 (98.2)	5 (99.5)	1 (99.7)	1 (100)					0.03	0.06
BSI (201)		3 (1.5)	29 (15.9)	87 (59.2)	65 (91.5)	14 (98.5)	3 (100)							0.03	0.06
CSF (194)		7 (3.6)	24 (16.0)	75 (54.6)	73 (92.2)	11 (97.9)	2 (99.0)	1 (99.5)	1 (100)					0.03	0.06
POS (395)			4 (1.0)	17 (5.3)	72 (23.5)	172 (67.1)	109 (94.7)	19 (99.5)	2 (100)					0.12	0.25
BSI (201)			2 (1.0)	5 (3.5)	40 (23.4)	90 (68.2)	60 (98.0)	4 (100)						0.12	0.25
CSF (194)			2 (1.0)	12 (7.2)	32 (23.7)	82 (66.0)	49 (91.2)	15 (99.0)	2 (100)					0.12	0.25
ISA (153)^a^	3 (2.0)	6 (5.9)	24 (21.6)	66 (64.7)	29 (83.7)	19 (96.1)	4 (98.7)	2 (100)						0.03	0.12
BSI (70)	2 (2.9)	3 (7.1)	5 (14.3)	32 (60)	15 (81.4)	9 (94.3)	3 (98.6)	1 (100)						0.03	0.12
CSF (83)	1 (1.2)	3 (4.8)	19 (27.7)	34 (68.7)	14 (85.5)	10 (97.6)	1 (98.8)	1 (100)						0.03	0.12

AMB, amphotericin B. FLC, fluconazole; VRC, voriconazole; POS, posaconazole; ISA, isavuconazole.

BSI, bloodstream infection; CSF, cerebrospinal fluid.

^a^ 153 isolates of *Cryptococcus* species were submitted for determination of isavuconazole MIC, between 2017 and 2020.

**TABLE 2:** Distributions of minimum inhibitory concentrations (MICs) of 93 *Cryptococcus* isolates cultured from sources other than blood and cerebrospinal fluid, tested against key antifungals. Isolates were obtained from hospitalized patients with cryptococcosis worldwide, based on data from the antifungal Antimicrobial Testing Leadership and Surveillance database between 2010 and 2020. A subset of *Cryptococcus* isolates (*n* = 31) was submitted between 2017 and 2020 for determination of isavuconazole MICs. Numerals in parentheses indicate the cumulative percentage of tested *Cryptococcus* isolates inhibited at each respective MIC (mg/L) for a given antifungal agent.

Agent (no. of isolates tested)	No. (cumulative percentage) of isolates with indicated MIC values (mg/L)														MIC_90_ (mg/L)
	0.008	0.015	0.03	0.06	0.12	0.25	0.5	1	2	4	8	16	32	64	
AMB (93)					1 (1.1)	1 (2.2)	35 (39.8)	55 (98.9)	1 (100)						1
FLC (93)						1 (1.1)	4 (5.4)	14 (20.4)	34 (57.0)	33 (92.5)	7 (100)				4
VRC (93)	2 (2.2)	14 (17.2)	43 (63.4)	24 (89.2)	9 (98.9)	1 (100)									0.12
POS (93)		1 (1.1)	4 (5.4)	15 (21.5)	38 (62.4)	30 (94.6)	5 (100)								0.25
ISA (31)		8 (25.8)	9 (54.8)	9 (83.9)	4 (96.8)	1 (100)									0.12

AMB, amphotericin B. FLC, fluconazole; VRC, voriconazole; POS, posaconazole; ISA, isavuconazole.

For AMB, the MIC distribution culminates in 2 mg/L, with an MIC_90_ of 1 mg/L. In contrast, the MIC distribution extends to higher values to FLC, with a 100% inhibition rate observed at 16 mg/L (MIC_90_ value of 4 mg/L for FLC). The MIC distribution of *Cryptococcus* isolates for the newer triazole antifungals—VRC, POS, and ISA—shows 100% inhibition at significantly lower concentrations compared to FLC. Specifically, VRC and POS achieve full inhibition at 1 mg/L (with MIC_90_ values of 0.06 and 0.25 mg/L, respectively), while ISA achieves full inhibition at 0.5 mg/L (with an MIC_90_ value of 0.12 mg/L).

The ECVs of *Cryptococcus* species for antifungal agents discussed in this study are summarized in Table 3.

**TABLE 3:** Epidemiological cutoff values (ECV, mg/L) of different *Cryptococcus* species against key antifungal agents discussed in this investigation.

*Cryptococcus* species, antifungal agents, and the respective ECVs (mg/L)	Amphotericin B	Fluconazole	Voriconazole	Posaconazole	Isavuconazole
*C. neoformans* var. *neoformans, and C. neoformans* var. *grubii*	1	4	0.5	0.5	0.25
*C. gattii species complex*	0.5	8	1	0.5	0.25

The non-WT proportions among *Cryptococcus* isolates causing BSI/MME in the 2010–2020 antifungal ATLAS database were 9.1% for FLC, 0.3% for VRC, 0.5% for POS, 1.3% for ISA, and 2.0% for AMB, respectively, based on ECVs published in the PubMed literature.

### MIC distributions of various significant *Cryptococcus* species causing BSI/MME to key antifungals

Table 4 presents MIC values for isolates of the three predominant *Cryptococcus* species causing BSI/MME: *C. neoformans* (111 isolates), *C. grubii* (270 isolates), and *C. gattii* (11 isolates), against the evaluated antifungal agents.

**TABLE 4:** Distributions of minimum inhibitory concentrations (MIC) of isolates of three predominant *Cryptococcus* species, comprising *Cryptococcus neoformans* var. *neoformans* (*n* = 111), *C. neoformans* var. *grubii* (*n* = 270), and *Cryptococcus gattii* species complex (*n* = 11) cultured from blood or cerebrospinal fluid, tested against key antifungals. Isolates were collected from hospitalized patients with cryptococcosis worldwide, based on data from the antifungal Antimicrobial Testing Leadership and Surveillance database between 2010 and 2020. Isolates of *C. neoformans* var. *neoformans* and *C. neoformans* var. *grubii* were submitted for determination of isavuconazole MICs between 2017 and 2020. Numerals in parentheses indicate the cumulative percentage of *Cryptococcus* isolates inhibited at each respective MIC (mg/L) of a given antifungal agent.

Agents and species	No. (cumulative percentage) of isolates with indicated MIC values (mg/L)												
	0.004	0.008	0.015	0.03	0.06	0.12	0.25	0.5	1	2	4	8	16
Amphotericin B													
*C. neoformans* var. *neoformans*						1 (1.0)	12 (11.7)	43 (50.5)	55 (100)				
*C. neoformans* var. *grubii*							5 (1.9)	99 (38.5)	163 (98.9)	3 (100)			
*C. gattii* species complex							1 (9.1)	5 (54.5)	5 (100)				
Fluconazole													
*C. neoformans* var. *neoformans*							1 (1.0)	7 (7.2)	26 (30.6)	30 (57.7)	30 (84.7)	15 (98.2)	2 (100)
*C. neoformans* var. *grubii*								4 (1.5)	28 (11.9)	109 (52.2)	111 (93.3)	17 (99.6)	1 (100)
*C. gattii* species complex							1 (9.1)			4 (45.5)	5 (90.9)	1 (100)	
Voriconazole													
*C. neoformans* var. *neoformans*		7 (6.3)	27 (30.6)	39 (65.8)	32 (94.6)	6 (100)							
*C. neoformans* var. *grubii*		2 (0.7)	26 (10.4)	120 (54.8)	99 (91.5)	17 (97.8)	4 (99.3)	1 (99.6)	1 (100)				
*C. gattii* species complex		1 (9.1)		3 (36.4)	5 (81.8)	2 (100)							
Posaconazole													
*C. neoformans* var. *neoformans*			3 (2.7)	4 (6.3)	26 (29.7)	43 (68.5)	23 (89.2)	10 (98.2)	2 (100)				
*C. neoformans* var. *grubii*			1 (0.4)	13 (5.2)	46 (22.2)	124 (68.1)	80 (97.8)	6 (100)					
*C. gattii* species complex						3 (27.2)	5 (72.7)	3 (100)					
Isavuconazole													
*C. neoformans* var. *neoformans* (*n* = 24)	3 (12.5)	3 (25)	5 (45.8)	9 (83.3)	4 (100)								
*C. neoformans* var. *grubii* (*n* = 122)		3 (2.5)	19 (18.0)	56 (63.9)	25 (84.4)	15 (96.7)	3 (99.2)	1 (100)					
*C. gattii* species complex (*n* = 5)						4 (80)	1 (100)						

AMB exhibits a potent inhibitory effect across all variants of *Cryptococcus* species, with notably lower MICs for *C. neoformans*. FLC is shown to reveal an intriguing susceptibility pattern. *C. grubii* isolates exhibit a lower MIC distribution to FLC compared to *C. neoformans*, with statistical significance (*P* = 0.009).

A distinct susceptibility profile emerges for VRC and ISA. *C. neoformans* isolates displayed significantly lower MICs for these two agents when compared to *C. grubii* (both *P* values <0.005). Conversely, isolates of *C. gattii* (*n* = 11) tended to exhibit higher MIC values to three triazoles, especially for POS and ISA. Consequently, variability in triazole susceptibility was observed among different *Cryptococcus* species.

### Correlation analyses between MIC distributions of studied invasive *Cryptococcus* isolates for FLC, VCZ, POS, and ISA

**FIGURE 1:**
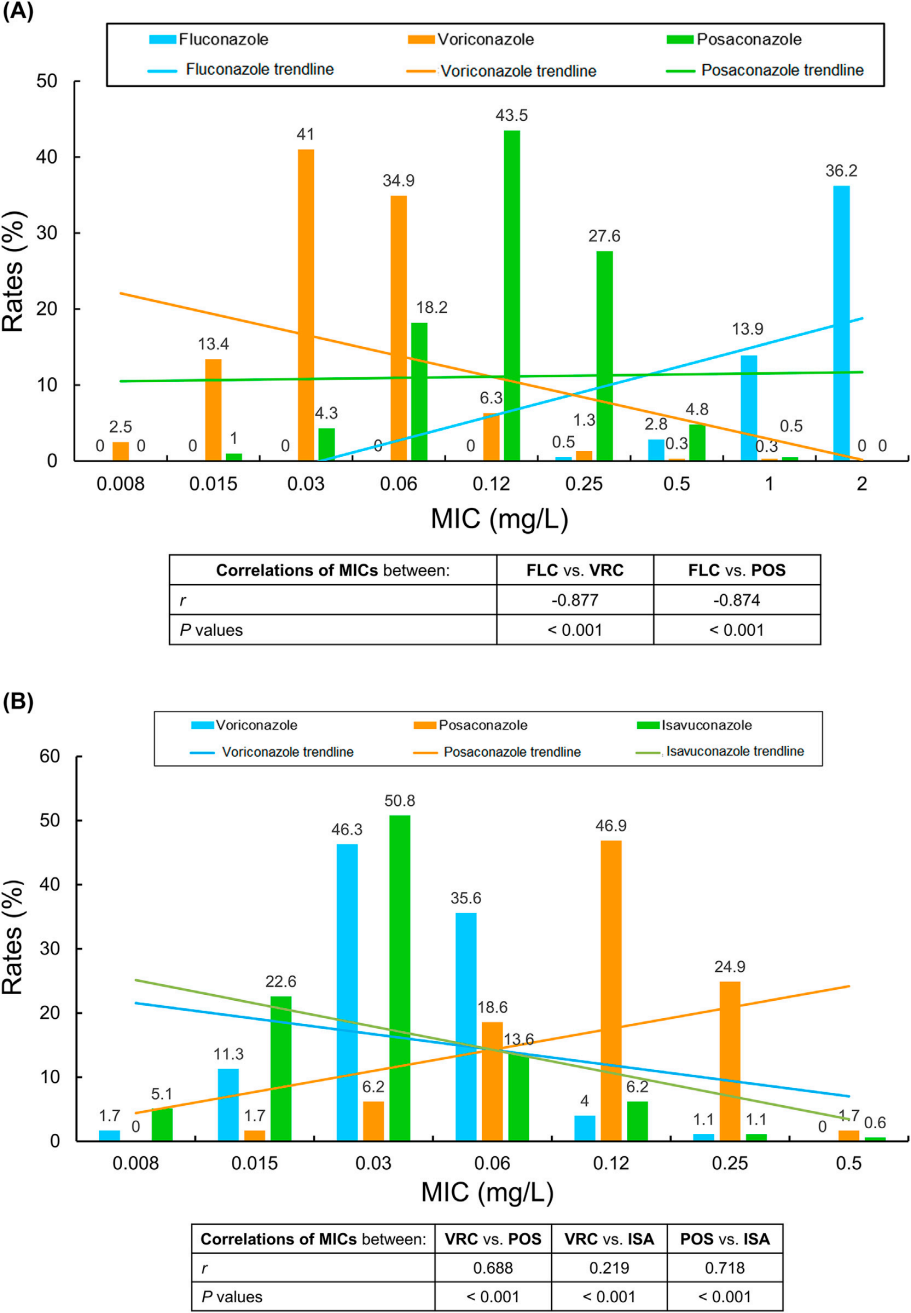
**(A,B)** Correlation trends between minimum inhibitory concentration (MIC) distributions for global *Cryptococcus* isolates collected between 2010 and 2020 **(A)** and between 2017 and 2023 **(B)** associated with bloodstream infections and meningitis/meningoencephalitis. MICs were evaluated against four azole antifungals: fluconazole, voriconazole, posaconazole, and isavuconazole. Spearman correlation analyses were conducted to assess relationships between agents. Linear trendlines are shown for all pairwise comparisons MIC, minimum inhibitory concentration. FLC, fluconazole. VRC, voriconazole. POS, posaconazole. ISA, isavuconazole.

Spearman rank correlation was considered scientifically more appropriate and statistically valid than Pearson’s correlation analysis due to the non-normal distribution for all antifungal MIC data in this investigation. Accordingly, we analyzed the MICs of 395 invasive *Cryptococcus* isolates for FLC and VRC, POS using Spearman correlation analysis. A strong negative correlation was observed between FLC and VRC (r = −0.877, *P* < 0.001), as well as between FLC and POS (r = −0.874, *P* < 0.001) (Figure 1A).

In contrast, among 177 invasive *Cryptococcus* isolates globally collected between 2017 and 2023, a moderately strong positive correlation was observed between VRC and POS (*r* = 0.688, *P* < 0.001), along with a weaker correlation between VRC and ISA (*r* = 0.219, *P* < 0.001), and a strong correlation between POS and ISA (*r* = 0.718, *P* < 0.001) using Spearman correlation analysis as well (Figure 1B). Furthermore, using non-parametric tests to evaluate the MIC distributions of these global invasive *Cryptococcus* isolates for the three newer triazoles, we found that POS MICs were significantly right-skewed, followed by VRC and then ISA. The respective MIC_90_ values were 0.25, 0.06, and 0.12 mg/L (all *P* values <0.001).

### Other noteworthy points related to PK-PD indices for key antifungals against *Cryptococcus* isolates

Although the MIC distributions of global *Cryptococcus* isolates for key antifungal agents were analyzed in the preceding section, their relationships with PK-PD indices relevant to clinical prescription for effective treatment of *Cryptococcus* infections that caused BSI/MME required further exploration. In the following section, we attempt to analyze the application of antifungal MIC distributions alongside associated PK–PD parameters to guide optimal antifungal dosing for the management of *Cryptococcus* infections causing BSI and MME, based on appropriate evidence selected from the PubMed literature database.

## Discussion

The strong negative correlations observed between FLC and VRC/POS MICs (Figure 1A) suggest that possible resistance mechanisms to FLC—such as mutations in *ERG11* (encoding lanosterol 14α-demethylase [i.e., CYP51]) and efflux pump overexpression—may not confer cross-resistance to newer triazoles like VRC and POS. Moreover, chromosomal plasticity, particularly whole-chromosome aneuploidy (notably chromosome one disomy in *C. neoformans*), can emerge rapidly under triazole pressure and markedly increase FLC MICs, facilitating *Cryptococcus* adaptation to FLC and highlighting in-host evolution driven by genome plasticity (Sionov et al., 2010).

The parallel trend in VRC and POS MIC distributions (Figure 1B) indicates possible co-selection of resistance upon exposure, implying that empirical use of either agent may be less reliable without MIC-guided therapy.

Structurally, POS contains a rigid triazole core and a bulky hydrophobic side chain, whereas ISA features a bulky isobutyryloxy side arm attached to its core, which greatly enhances the orientation of its triazole ring and enables tighter binding to fungal CYP51 (Datta et al., 2013). Differences in CYP51 binding affinity between ISA and VRC/POS to *Cryptococcus* isolates suggest ISA’s ability to evade common resistance mechanisms of other azoles, such as overexpression of the ABC transporter AFR1 (Arastehfar et al., 2020), which actively extrudes many triazoles from the fungal cells, thereby lowering their intracellular concentrations; and point mutations like Y145F and G484S in the *ERG11* gene (Sionov et al., 2012), which alter the CYP51 binding pocket and reduce the affinity of many triazoles, particularly for VRC and POS, to non-WT *Cryptococcus* isolates. As a result, cross-resistance between ISA and other triazoles cannot be reliably inferred.

These findings may have therapeutic implications. The distinct susceptibility profile of ISA suggests that it could serve as an alternative treatment option for non-WT *Cryptococcus* strains exhibiting elevated MICs to FLC, VRC, and POS. ISA or LAmB should be strongly considered for the treatment of systemic cryptococcosis in patients with recent exposure to VRC or POS.

**FIGURE 2:**
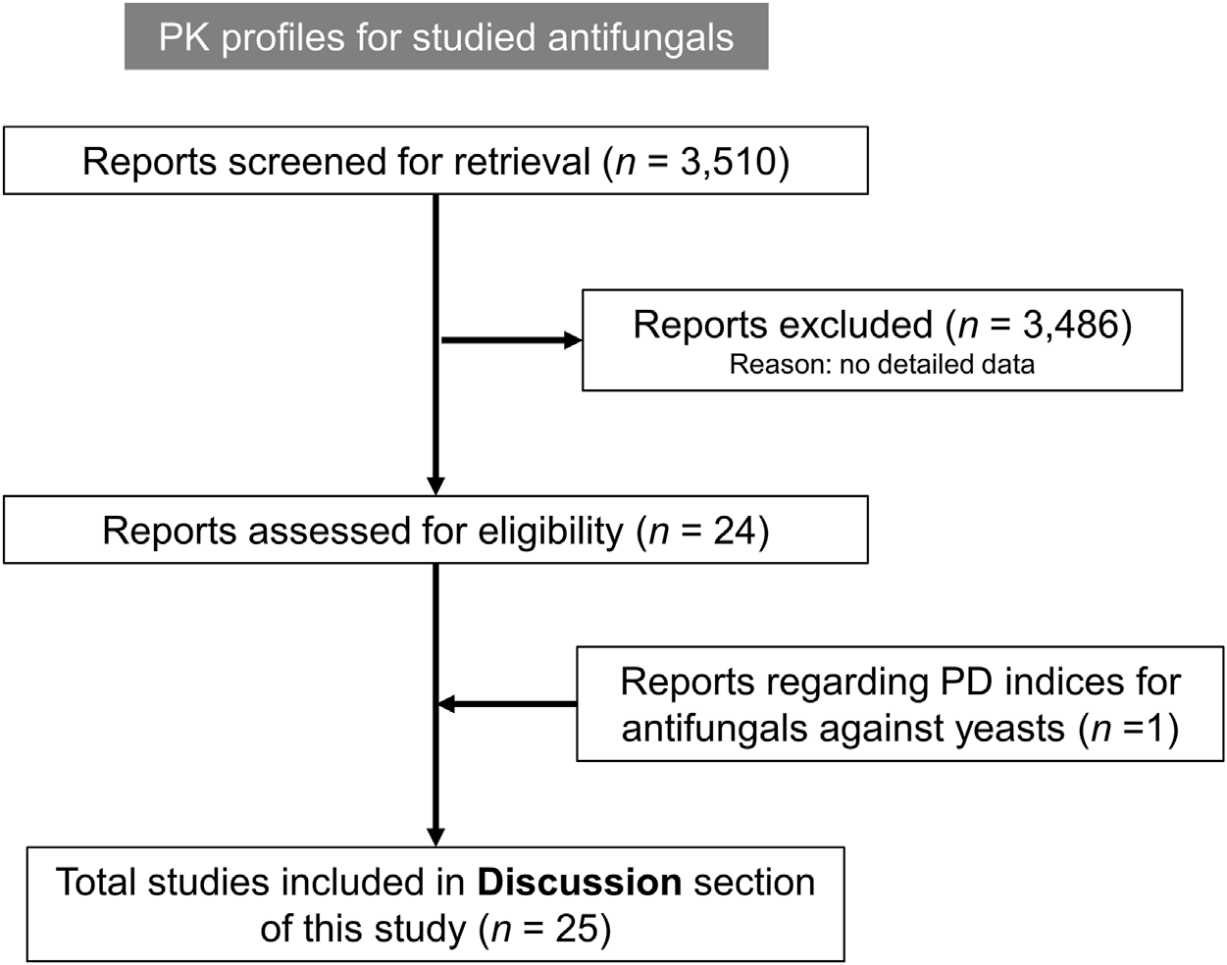
Flow diagram illustrating the number of PubMed literature reports that were screened and excluded during the analyses of pharmacokinetic (PK) profiles for the antifungal agents under investigation, and pharmacodynamic (PD) indices for the studied antifungals against yeasts.

In this study, we identified dosing strategies optimized for the effective treatment of invasive *Cryptococcus* infections by leveraging antifungal MIC data from the ATLAS database, documented ECVs, and published PK–PD parameters. Additionally, as illustrated in Figure 2, we cautiously excluded 3,486 studies that did not report the detailed methods to generate the PK data for antifungals, or clinical outcomes for patients with cryptococcosis receiving antifungal therapy.

The published ECVs for FLC against *Cryptococcus* species range from 4 mg/L (for *C. neoformans* and *C. grubii*) to 8 mg/L (for *C. gattii*) (Espinel-Ingroff et al., 2012a; Jean et al., 2024; Lockhart et al., 2012). Among the invasive *Cryptococcus* isolates evaluated, approximately 91% had FLC MICs below 4 mg/L and were classified as WT strains (Table 1).

For FLC, we screened 1,264 studies to investigate its PK profile. Based on the PK data for FLC (Arredondo et al., 1994; Buijk et al., 2001; Nau et al., 2010; Santos et al., 2016) (Table 5), the free-drug area under-the-concentration-time curve from zero to 24 h (AUC_0-24_) is estimated to range from 191.7 μg·h/mL to 375.4 μg·h/mL in CSF, and from 157.6 μg·h/mL to 226.1 μg·h/mL in brain tissue for patients receiving 400 mg of FLC daily. Accordingly, regrowth of *Cryptococcus* isolates residing in brain tissue with an MIC of ≤4 mg/L to FLC could be effectively inhibited in patients with CNS cryptococcosis receiving FLC at 400 mg/day.

**TABLE 5:** Pharmacokinetic (PK) profiles and pharmacodynamic (PD) indices of antifungal agents (triazoles, and liposomal amphotericin B) against yeasts (*Cryptococcus* species) that were discussed in this antifungal Antimicrobial Testing Leadership and Surveillance survey.

Antibiotics, dosages (PD indices against yeasts)	PK profiles and molecular weights				
	Total-drug AUC_0-24_ (μg•h/mL) in plasma, or C_max_ (doses of administration)	Half-life (h)	Protein binding (%)	Molecular weight (g/mol)	(1) Ratios of CSF, or brain-to-plasma [antifungal] (meaning antifungal concentration), or (2) Area under the concentration-time curve from 0 to 24 h at the infection site (AUC_0–24,site_), or (3) Cerebral or CSF concentration ([drug]_brain_, [drug]_CSF_)
FLC [AUC_0-24_/MIC ≥25 at the infection site (Li et al., 2010)]	336–482, with a mean of 409 (400 mg qd) (Buijk et al., 2001)	30 (Felton et al., 2014)	12.5–22.9 (Arredondo et al., 1994)	305 (Felton et al., 2014)	1.0.74–0.89, for [FLC]_CSF_/[FLC]_plasma_ ratio, calculated in terms of total concentration (Nau et al., 2010) 2.0.469, for the ratio of AUC_0-24,cerebrum_/total-drug AUC_0-24,plasma_ (Santos et al., 2016)
VRC [AUC_0-24_/MIC ≥25 at the infection site (Li et al., 2010)]	59.0; and for free-drug, 24.8 (6 mg/kg bid on day 1, then 4 mg/kg bid since day 2) (Xu et al., 2021)	6.1 (Jean et al., 2022)	58 (Jean et al., 2022)	349 (Felton et al., 2014)	1.0.46, for the ratio of [VRC]_CSF_/[VRC]_plasma_, calculated in terms of total-drug concentration (Nau et al., 2010) 2.1.9 μg/g on a dosage of 4 mg/kg bid (Elter et al., 2006)
ISA [AUC_0-24_/MIC ≥25 at the infection site (Li et al., 2010)]	97.0 (372 mg every 8 h for 6 doses, and 372 mg once daily since day 3) (Desai et al., 2016)	85–117 (Jean et al., 2022)	>99 (Jean et al., 2022)	438 (Felton et al., 2014)	1.1.8, for the ratio of [ISA]_brain_/[ISA]_plasma_ (rat model) (Wiederhold et al., 2016) 2.0.02–0.04, for the ratio of [ISA]_CSF_/[ISA]_plasma_ in humans (Kovanda et al., 2019)
POS [AUC_0-24_/MIC ≥25 at the infection site (Li et al., 2010)]	17.2 ± 14.8 (multiple doses of 400 mg bid) (Li et al., 2010)	24–27 (Jean et al., 2022)	>98 (Jean et al., 2022)	700 (Felton et al., 2014)	1. Poor (both <0.01) penetration ratios in terms of plasma-to-CSF and plasma-to-brain concentration for POS (Felton et al., 2014)
LAmB [C_max, site_/MIC ≥4 (Li et al., 2010)]	Total-drug C_max_, 5.17 (+1.89) [multiple doses of 4.55 + 0.23 mg/kg/day for 6–7 days) (Jean et al., 2022)]	400 (Jean et al., 2022)	98 (Jean et al., 2022)	924 (Felton et al., 2014)	1. (1.99 + 0.33) – (2.32 + 0.51) μg/g for [LAmB]_brain_, on a dosage of 5 mg/kg/day for >7 doses (rabbit model) (Lee et al., 1994) 2.0.024 + 0.001 μg/mL, for [LAmB]_CSF_ on 5 mg/kg/day (rabbit model) (Lee et al., 1994)

[Drug]_sample_, the concentration of a given antifungal at the specific body (infection) site. FLC, fluconazole; VRC, voriconazole; POS, posaconazole; ISA, isavuconazole. LAmB, liposomal amphotericin B. PK, pharmacokinetic; PD, pharmacodynamic. AUC_0–24_, area under the concentration-time curve from 0 to 24 h. C_max_, maximal concentration. MIC, minimum inhibitory concentration. qd, once daily. bid, twice daily. CSF, cerebrospinal fluid.

Similar to the global WT rate of 90.9% (Jean et al., 2024), approximately 91% of worldwide *Cryptococcus* isolates in the CSF exhibited FLC MICs ≤4 mg/L. Additionally, data from the 2010–2020 antifungal ATLAS database indicate that the MIC distribution of invasive *Cryptococcus* isolates against FLC was statistically shifted toward lower values in the Asia–Western Pacific region and Europe (MIC_90_ = 4 mg/L), compared to higher values in North and Latin America (MIC_90_ = 8 mg/L) (Jean et al., 2024).

Because FLC concentrations in blood and CSF correlate strongly with daily dose in humans (Schiave et al., 2018), increasing the maintenance dose of FLC to at least 800 mg/day may be necessary for effective treatment of patients with CNS cryptococcosis during the induction phase—particularly in regions outside the Americas. For invasive *Cryptococcus* infections in North and Latin America, and specific genotypes of *C. gattii* isolates in China and Colombia (Firacative and Escandón, 2021; Hagen et al., 2010; Xu et al., 2021), increasing the FLC maintenance dose up to 1,200 mg/day, adding 5-flucytosine (5-FC), or switching to VRC (see below) during the induction phase appears to be warranted. This dosage rationale is consistent with previous investigations (Schiave et al., 2018; Sudan et al., 2013).

For VRC, the published ECVs for *Cryptococcus* species reportedly ranged from 0.5 mg/L (for *C. neoformans* and *C. grubii*) to 1 mg/L (for *Cryptococcus gatti*) (Espinel-Ingroff et al., 2012a; Jean et al., 2024; Lockhart et al., 2012). The majority of *Cryptococcus* isolates collected in the 2010–2020 ATLAS project exhibited MIC ≤0.5 mg/L to VRC (100% for BSI isolates and 99.5% for CSF isolates) (Table 1).

For effective treatment of yeast infections, an AUC/MIC ratio greater than 25 is generally recommended for azoles (Li et al., 2010). If the MICs of *Cryptococcus* isolates to VRC approach the ECV (1 mg/L), achieving an adequate AUC of ≥25 μg h/mL at the infection site becomes critical. For VRC, we screened 1,130 studies to investigate its PK profile. Integrating the human PK profiles from important literature regarding administration of standard VRC dosage (Table 5) (Al-Saigh et al., 2012; Elter et al., 2006; Felton et al., 2014; Hope, 2012), VRC appears to be effective in inhibiting the growth of *Cryptococcus* isolates in the CNS, even in the absence of pronounced meningeal inflammation.

Anecdotal case reports have also demonstrated the efficacy of VRC after other antifungal therapies failed in the treatment of cryptococcosis (Bandettini et al., 2009; Carbonara et al., 2009; Chang et al., 2010; Shen et al., 2008). However, therapeutic drug monitoring (TDM) for VRC is warranted during its administration to avoid adverse effects (e.g., hepatotoxicity, visual disturbances, and neurotoxicity), as significant variations in serum levels can occur due to multiple influencing factors.

The published ECV for POS against major *Cryptococcus* species is 0.5 mg/L (Espinel-Ingroff et al., 2012a; Jean et al., 2024; Lockhart et al., 2012). Similar to VRC, 99.5% of invasive *Cryptococcus* isolates collected in the 2010–2020 ATLAS project were categorized as WT strains for POS (Table 1).

For POS, we screened 595 studies to investigate its PK profile. Although the 2024 global cryptococcosis treatment guidelines list POS as an optional alternative to FLC (Chang et al., 2024), the molecular weight of POS (approximately 700 g/mol) is significantly higher than that of other triazoles (Felton et al., 2014). This characteristic may partly explain the extremely low concentrations of POS in brain tissue and CSF (Felton et al., 2014). These PK limitations are consistent with the findings from a case series by Pitisuttithum et al., which enrolled 29 patients with cryptococcal meningitis receiving POS therapy (Pitisuttithum et al., 2005). The series reported a clinical failure rate of 51.7% (15/29). Among the 14 patients with successful outcomes, 6 (42.9%) still tested for positive India ink staining in the CSF, and one patient remained serum cryptococcal antigen-positive (Pitisuttithum et al., 2005). Accordingly, POS appears to be a suboptimal option for managing cryptococcosis involving the CNS, especially when compared to other triazoles (Arredondo et al., 1994). It is noteworthy that POS is predominantly metabolized by the CYP3A4 enzyme. TDM for immunosuppressants is essential to optimize their efficacies and minimize toxicities in immunocompromised hosts.

The published ECV for ISA against major *Cryptococcus* species is 0.25 mg/L (Espinel-Ingroff et al., 2015; Jean et al., 2024). Also similar to VRC and POS, 98.7% of the studied *Cryptococcus* isolates exhibited MIC below 0.25 mg/L for ISA (Table 1).

For ISA, we screened 234 studies to investigate its PK profile. ISA displays highly variable PK in human plasma (Desai et al., 2016). Despite these data, adjustment of the ISA maintenance dosage is usually not needed.

Notably, ISA achieves adequate concentrations in brain tissue (Wiederhold et al., 2016) but demonstrates poor penetration into the CSF, with plasma-to-CSF concentration ratios ranging from 0.02 to 0.04 in a rabbit model (Kovanda et al., 2019). This poses a significant challenge for its use in cryptococcal MME unless supported by another antifungal agent. Additionally, similar to POS, ISA is primarily metabolized by the CYP3A4 enzyme and, to a lesser extent, *via* glucuronidation pathways. TDM for key immunosuppressants is essential in immunocompromised hosts. Nevertheless, ISA may be preferred over VRC in elderly patients for antifungal treatment.

A small case series by Thompson et al. included five patients with cryptococcal meningitis treated with standard ISA dosing (372 mg every 8 h for six doses, followed by 372 mg once daily from day 3 onward) for durations ranging from 11 to 181 days. This study reported that there was complete success in only one case, partial success in three cases, and a fatal outcome in one case—despite sterile CSF cultures (Thompson et al., 2016). These findings further raise concerns regarding the clinical efficacy of ISA for the treatment of cryptococcosis, particularly in CNS infections.

**FIGURE 3:**
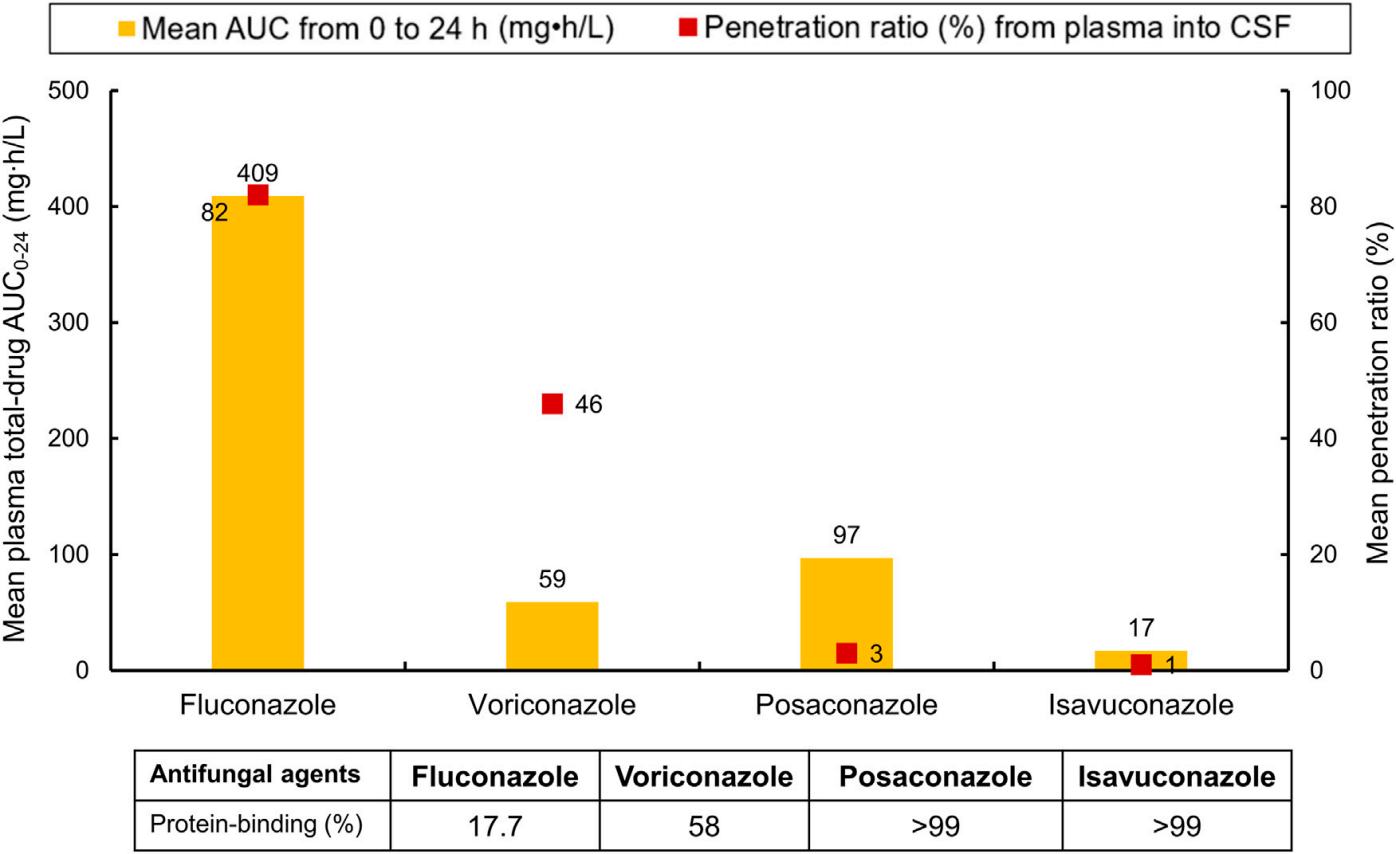
Important pharmacokinetic parameters (including mean total-drug plasma AUC_0–24_, protein-binding rates, and ratios of plasma-to-CSF penetration) for the four triazoles. AUC_0–24_, area under-the-concentration-time curve from zero to 24 h. CSF cerebrospinal fluid. Dosages required to achieve the respective mean AUC_0–24_ were as follows: fluconazole, 400 mg once daily; voriconazole, 6 mg/kg every 12 h on day 1, followed by 4 mg/kg every 12 h from day 2 onward; posaconazole, 400 mg twice daily; isavuconazole, 372 mg every 8 h for six doses, followed by 372 mg once daily from day 3 onward.

A summary of key PK parameters—including mean total-drug plasma AUC_0–24_, protein-binding rates, and ratios of plasma-to-CSF penetration—for the four triazoles is illustrated in Figure 3.

For AMB, the published ECVs for *Cryptococcus* species range from 0.5 mg/L for *C. gattii* to 1 mg/L for *C. neoformans* and *C. grubii* (Espinel-Ingroff et al., 2012b; Jean et al., 2024). Approximately 43% of *Cryptococcus* isolates had MICs ≤0.5 mg/L, and 99% had MICs ≤1 mg/L to AMB (Table 1). Additionally, 55% of the studied *C. gattii* isolates (6/11) were categorized as WT strains, in contrast to 99%–100% of the *C. neoformans* and *C. grubii* isolates, which were WT (Table 4). Notably, the potential to AMB deoxycholate to achieve adequate CSF concentrations is constrained by its toxicity (Bekersky et al., 2002).

Over the past 2 decades, LAmB has been increasingly favored for the treatment of cryptococcal MME due to its reduced toxicity profiles (Iyer et al., 2021; Jarvis et al., 2019; Jarvis et al., 2022). Additionally, LAmB shows non-inferior efficacy to AMB deoxycholate for treatment of high fungal burden cases, such as disseminated cryptococcosis or severe pulmonary disease (Jarvis et al., 2022; Kimuda et al., 2025).

For LAmB, we screened 287 studies to investigate its PK profile. The useful PK profiles were clearly documented in a few investigations (Jean et al., 2022; Lee et al., 1994; O'Connor et al., 2013). The optimal PD index of AMB against yeasts is the ratio of the maximal concentration (C_max_) to MIC (C_max_/MIC), with a target value of ≥4 at the site of infection (Li et al., 2010).

Jarvis et al. demonstrated that a single high-dose administration of 10 mg/kg LAmB on day 1 is required in combination with maintenance antifungals with good CSF penetration (e.g., FLC 1200 mg/day and 5-FC 100 mg/kg/day) to achieve optimal CSF clearance rates of *Cryptococcus* (−0.40 log_10_ cfu/mL/day) in HIV patients with cryptococcal MME (Jarvis et al., 2022). Although data on the efficacy of a single high-dose LAmB in SOT recipients and other cryptococcal disease syndromes are lacking, a single 10 mg/kg LAmB therapy for cryptococcal BSI/MME is anticipated to significantly reduce the health-economic burden in low- and middle-income countries compared to a 3–4 mg/kg LAmB daily dosage for 7–14 days, and thus is worthy of broader consideration.

This survey of antifungal MIC distributions for *Cryptococcus* isolates has several limitations. First, the PD target used for *Cryptococcus* in this study was extrapolated from *Candida* studies, which may not accurately reflect cryptococcal pathophysiology. Second, LAmB and the newer triazoles remain largely unavailable in many low-income countries, limiting generalizability of the findings. Third, the number of *C. gattii* isolates in this study was too small to draw reliable conclusions. Fourth, even with early diagnosis, low MIC levels against *Cryptococcus* isolates and apparently optimal PK-PD profiles for a specific antifungal may not guarantee favorable outcomes, particularly in patients with severe systemic cryptococcosis and compromised immune conditions. Therefore, further clinical studies are needed to validate these observations.

## Conclusion

In conclusion, all the newer triazoles (VRC, POS, ISA) demonstrated low MIC values against major *Cryptococcus* species. Both VRC and FLC achieve optimal CNS penetration. The combination of LAmB with FLC (1,200 mg daily) and 5-FC (100 mg/kg daily)–a cost-effective regimen–has been listed the standard induction–phase regimen. VRC exhibits superior PK characteristics for managing CNS cryptococcosis relative to ISA and POS, and appears to be a reasonable alternative to FLC during the induction treatment. However, due to substantial interindividual variability in PK profiles and notable drug-drug interactions, TDM for VRC is indicated for dose optimization. Additionally, ISA may represent a preferable option over POS during the consolidation phase of cryptococcosis treatment.

## Data Availability

The original contributions presented in the study are included in the article/supplementary material, further inquiries can be directed to the corresponding author Shio‐Shin Jean, jeanshioshin168@gmail.com.
